# Newly evolving pastoral and post-pastoral rangelands of Eastern Africa

**DOI:** 10.1186/s13570-020-00179-w

**Published:** 2020-11-16

**Authors:** Jeremy Lind, Rachel Sabates-Wheeler, Matteo Caravani, Luka Biong Deng Kuol, Deborah Manzolillo Nightingale

**Affiliations:** 1 Institute of Development Studies, University of Sussex, Falmer, Brighton, BN1 9RE UK; 2 World Food Programme, Rome, Italy; 3 Africa Center for Strategic Studies, National Defense University, Washington, D.C, USA; 4 Houston, USA

**Keywords:** Livelihoods, Poverty, Vulnerability, Population growth, Livestock marketing, Capital investment, Uncertainty

## Abstract

Over the past two decades, the rangelands of Eastern Africa have experienced sweeping changes associated with growing human populations, shifting land use, expanding livestock marketing and trade, and greater investment by domestic and global capital. These trends have coincided with several large shocks that were turning points for how rangeland inhabitants make a living. As livelihoods in the region’s rangelands transform in seemingly paradoxical directions, away from customary pastoralist production systems, greater insight is required of how these transformations might affect poverty and vulnerability. This article reviews the state of what is known regarding directions of livelihood change in the rangelands of Eastern Africa, drawing on case studies of structural change in five settings in the region. It considers the implications of long-term change, as well as the emergence of very different livelihood mixes in pastoral rangelands, for efforts to reduce poverty and vulnerability in these places.

## Introduction

While pastoralism remains the most productive use of most of Eastern Africa’s rangelands, customary pastoral production systems have come under immense pressure in recent decades. Several, seemingly paradoxical, dynamics define the challenge for securing livelihoods in a context of evolving pastoral and post-pastoral rangelands. We identify four such paradoxes. First, mobile and semi-mobile livestock keeping is the most productive activity in nearly all rangelands, yet, per capita livestock holdings have declined over a long period and continue to diminish across most parts of dryland Eastern Africa (Desta et al. 2008; Devereux 2006; Little et al. 2001; Lybbert et al. 2004; McCabe et al. 2010; Deng 2008). Livestock holdings per capita now fall far short of subsistence requirements for a large proportion of pastoralist populations. Second, commercialisation of the livestock sector and the export trade in live animals and carcasses has experienced significant growth, particularly in Ethiopia, but levels of poverty and vulnerability are worsening. The breadth and depth of vulnerability were evident during the 2011 drought crisis, which affected nearly 10 million people in Somalia, Kenya, Ethiopia and Djibouti, and again in 2020 when the region was buffeted by multiple crises, including devastating locust swarms and the COVID-19 pandemic. Crises past and present tipped many into a situation of acute food insecurity, leading to region-wide efforts to develop approaches to address vulnerability and support weakened livelihoods in pastoral areas. Third, the mobility of people with herds has greatly decreased, yet, the concomitant sedentarisation has been marked by the dispersion of households, with members migrating to towns, urban centres and beyond for work, social assistance and education. Fourth, perennial uncertainty in both climate and disease necessitates flexibility and adaptability, yet, rangelands are fragmenting as an increasing proportion of the land area (and particularly key grazing areas) are being enclosed for state (conservation) and private uses (crop production), limiting passage and livestock movements.

These paradoxical dynamics define the challenge for programming and interventions that endeavour to strengthen livelihoods in the rangelands. Dynamic change in rangeland Eastern Africa has resulted in differentiated outcomes geographically but also socially for the diverse inhabitants of these areas. Here, we present the results of a comprehensive review of evidence pertaining to key influences on and directions of change in livelihoods in Eastern Africa’s pastoral and post-pastoral rangelands, focussing on changes since 2000 when a regional drought crisis precipitated relief interventions at scale. The purpose of this is to identify the commonalities and differences in evolving livelihood mixes across different rangelands as well as the implications of these for strengthening responses to risk and uncertainty. Different livestock-based production systems have emerged in varying political-economic and socio-ecological settings in the drylands, underlining the importance of understanding trajectories of change that unfold in specific places. Today, varieties of pastoralism include (i) commercialised forms of livestock-keeping oriented to large domestic and regional export markets, (ii) smaller-scale livestock-keeping for subsistence and local marketing, combined with subsistence farming and other rural activities, (iii) the maintenance of very few small stock in and close to towns alongside the pursuit of various tasks for cash and (iv) customary pastoralism based on long distance movements, key resource use and maintaining a network of bond friendships through which to exchange livestock and labour as the basis for managing uncertainties.

**Fig. 1:**
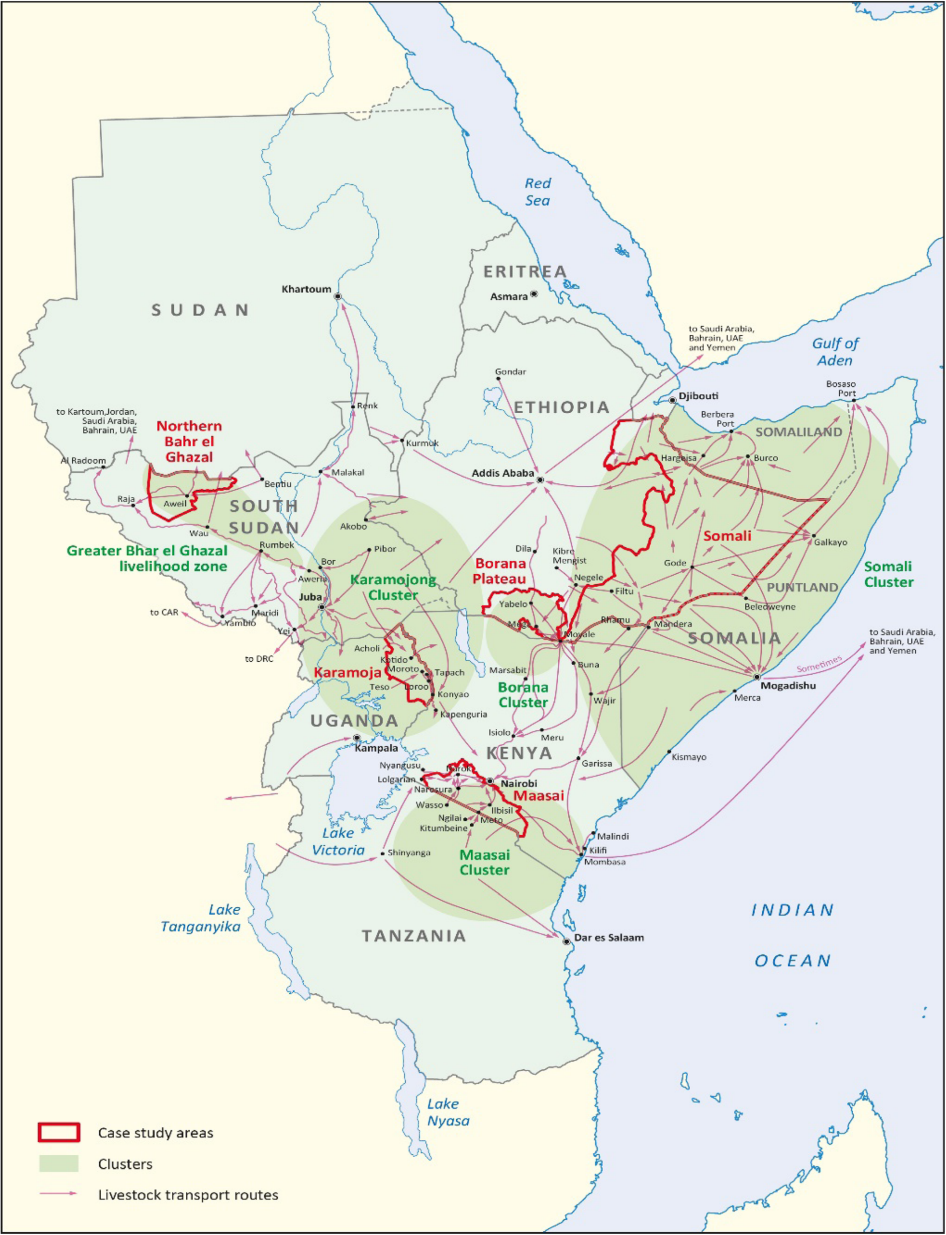
Rangeland contexts in Eastern Africa

The article focusses on five rangeland contexts (see Fig. 1): Kenya’s south Rift Valley, the Somali Region of Ethiopia, the Borana Plateau in southern Ethiopia, Karamoja in north-eastern Uganda and northern Bahr el Ghazal in South Sudan. These areas were purposely identified because they are emblematic of some of the emerging livelihood mixes in the region’s rangelands, which in turn connect with varying drivers of change as well as shifting profiles of poverty and vulnerability.

Through a comparative lens, we use evidence from these rangeland settings to examine trajectories of change as well as the main influences on livelihoods. The article is based on a comprehensive review and synthesis of existing secondary evidence and literature as well as detailed case studies of dynamics and changes in the five rangeland contexts. More than 400 documents were retrieved by formal literature search using several databases complemented by manual back-searching, as well as by snowballing techniques to identify additional literature. In addition, key informant interviews were carried out with experts on the different rangeland contexts and themes. These experts also provided grey literature and other documents that were not identified through desk-based searches. The remainder of this article draws on the findings and analysis of this extensive literature review and key informant interviews.^1^

## Change and divergence in livelihoods

Over time, livelihoods in the rangelands of Eastern Africa have been redefined as they have become increasingly bound into processes of state territorialisation, large-scale infrastructure and resource development, and regional trade and investment. These processes are unfolding alongside a steady rise in local capital in these areas and a shift in livestock holdings to those who are not mobile and who may not even reside in pastoral areas. Sweeping changes in land use, with many seeking to enclose bits of land and try their hand at farming, and the growth of towns and regional centres, signal wider transformations happening, as well (Aklilu et al. 2016; Coppock et al. 2018; Davies and Moore 2016; Greiner 2020). These processes and dynamics are apparent in several distinct, longitudinal trends in the ways that inhabitants of the rangelands make a living, as examined below. Still, even though multiple rather different ways of making a living have emerged alongside livestock-keeping, pastoralism remains the backbone of economic life in the rangelands. As Kratli and Swift (2014) note, the importance of pastoralist systems does not correlate with the numbers of people who actually remain in such households or livestock holdings.

**Fig. 2:**
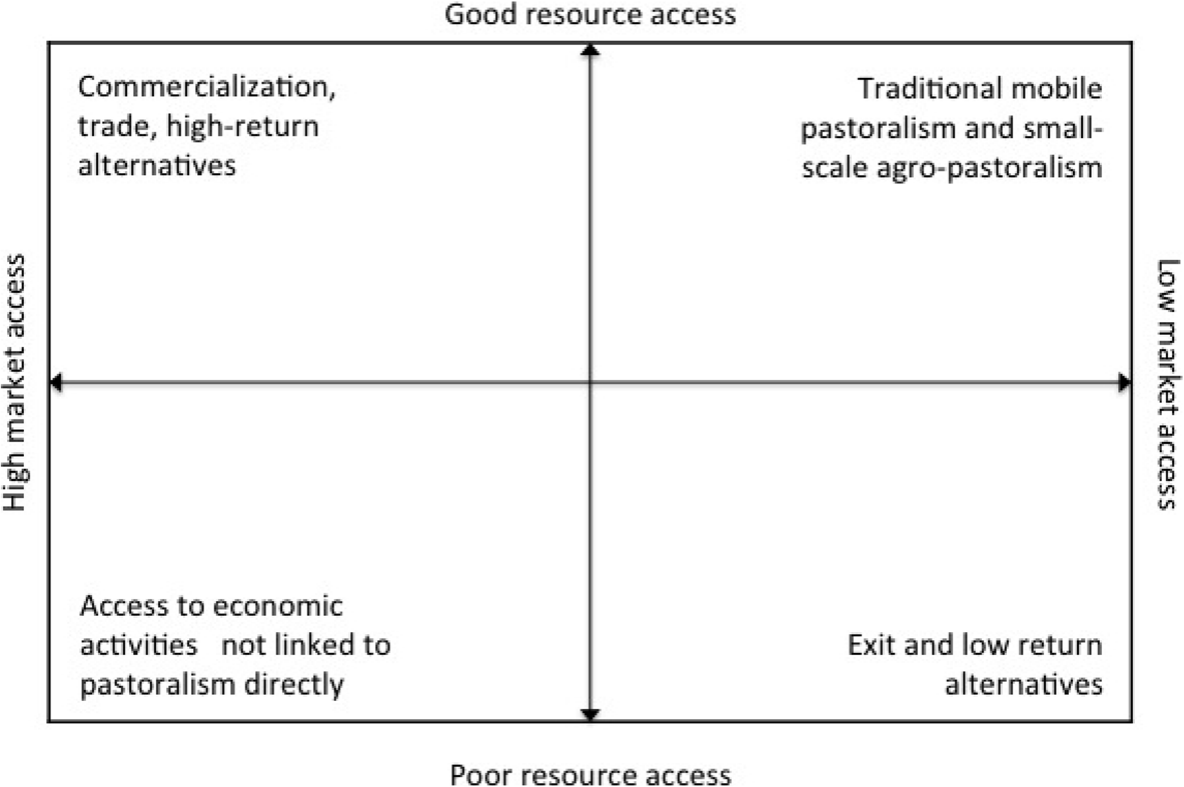
Livelihood pathways in rangeland Eastern Africa

This evolution in rangeland livelihoods in Eastern Africa is evident in our conceptual understanding of different pathways for how inhabitants of pastoral areas in the region make a living (Fig. 2), building on previous schemas developed by Catley et al. (2013), Dorward (2009), and Mushongah (2009). As with any conceptual framework, this conceals enormous variation and nuance in livelihood pathways across the region. Conceptual categorisations can essentialise very dynamic livelihoods, imposing an order and predictability on what are far more complex situations and directions of change. Further, as noted below, pastoralists move in and out of livestock-keeping over time; this brings its own complications in terms of mapping shifts in livelihoods when movement in one direction could be transitory, and quite possibly strategic to move back into pastoralism, even if a different form. However, categorisations of livelihood change have proven useful to policy thinking and applied research on the very different and diverging trajectories for inhabitants of pastoral areas. Some are *moving up* into commercialisation, regional and export livestock trades and other high-return economic activities; others are *moving out* into activities not linked to pastoralism directly but that may nonetheless be linked to livestock-keeping through various feedback loops and value-added diversification activities; some are *hanging in* traditional mobile pastoralism and small-scale agro-pastoralism, while many more are *dropping out* or exiting into a range of tasks for cash and other low-return economic activities. While these different trajectories are discussed below in turn, they closely entwine and are not clearly partitioned. For example, trends in privatisation and commodification of rangeland resources, which closely align with moves up into higher value livestock marketing and trade in some rangelands, are leading to more families dropping out. Thus, not only does more than one pathway exist in a single geographic setting, but also options and opportunities aligning with a particular pathway have costs and benefits for others following a different pathway.

Areas and people with good natural resource access and access to markets are *moving up*, because, among other things, they are able to maintain and sell livestock and their products as a successful business enterprise, commercialising the milk and livestock trade, selling in high export zones, creating private abattoirs and finding lucrative business opportunities along the livestock value chain. Pastoralists moving up are particularly evident in the high-export zones of Ethiopia’s eastern lowlands, which historically keep many cross-border trade and exchange relations with neighbouring areas of Somaliland, Puntland and Somalia (Catley and Aklilu 2013). Yet, the effects of this trade ripple outwards, extending into northern Kenya, where livestock keepers increasingly supply camels to the regional trade through Moyale-based traders (Mahmoud 2013). Profits from livestock commercialisation are spurring an assortment of high-return economic activities, seen in the establishment of private ranches where grazing is hired for a fee, to the establishment of private abattoirs (in areas of Somalia and Somaliland) to a rental market in small towns and urban centres in Karamoja, Borana and Isiolo (Stites et al. 2014; Korf et al. 2015; Coppock et al. 2018; Elliott 2020).

Others are *moving out* of customary livestock production into an assortment of value-added activities (such as processing and fattening) as well as non-livestock-based activities. It is involvement in commercialised forms of livestock keeping that distinguishes the successful from those who are moving out of customary pastoralism. Many diversified activities by those moving out are still connected to livestock-keeping such as the preparation and sale of hides and skins, the sale of fodder, the provision of transport and micro-dairying to supply milk to the populations of fast-growing rural towns and regional centres. Small town expansion, better connections with larger centres and the younger generation’s acceptance of non-traditional livelihoods are all enabling value-added diversification for those who may own few or any animals. Sedentarisation has presented new economic opportunities, especially for women through the sale of agricultural produce, milk and labour. In Karamoja towns, women pursue a range of market strategies including selling livestock for cash, micro-enterprises (selling food items—purchase maize, beans and cowpeas and then resell mixtures for 100Ush, 4 US cents in 2010 conversion per spoonful) and selling local beer (Stites and Huisman 2010: 8; Caravani 2019). The income women earn from brewing now dwarfs that which they receive from engaging in casual labour or selling firewood.

Areas and people with good resource access, to rangeland and water sources in particular, but who do not have high market access are *hanging in*, practising customary forms of pastoralism based on high mobility, banking on extended social ties and opportunistic use of key resource patches within the wider landscape. Those who are hanging in customary pastoral systems now tend to live in permanent base camp settlements, wherein women engage in livelihood activities that are both connected to and diverging from customary pastoral livelihoods. Rangeland dwellers who are hanging in are not bound to fail; indeed, the persistence of customary pastoralism in many parts of Eastern Africa demonstrates its resilience in the face of multiple uncertainties, not just climate but also those induced by political choices as well as economic and technological change. The economic life associated with customary pastoralism connects in many ways with those who are following other livelihood pathways. For example, pastoralist incomes are driving demand for construction, services, crop produce, small industry and natural products (Headey et al. 2012), thus contributing to improvements in incomes and livelihoods for those who are moving out of a livelihood centred on keeping livestock. Pastoralists are also supplying regional livestock trades, sustaining commercialisation for those who are moving up, even while the production logic for most who are hanging in continues to be breeding and milk production.

When a pastoralist’s herd is no longer viable due to lack of good resource access, the household exits pastoralism, or *drops out*, at which point its members seek productive activities not directly linked to herding. Little et al. (2001) found that for the poorest herders, unskilled waged labour and petty trade were the most common non-pastoral options. There is of course nothing new about individuals from pastoralist backgrounds moving away from livestock-keeping in search of alternatives as their herds diminish. Yet, the context in which people leave pastoralism is qualitatively different today, as processes of rangeland fragmentation have constrained access to key resources as well as created barriers to mobility, thereby making it harder to ‘move back’ into pastoralism. A sizeable and growing proportion of the population in rangelands is chronically vulnerable and lacks clear alternatives to livestock-keeping, much less options to return. Increasing numbers of households in the rangelands have exited customary pastoralism without experiencing any advantages that could be gained from positive diversification—such as those that are emerging in towns and urban centres (Caravani 2019; Desta et al. 2008; Headey et al. 2012). Many are destitute and survive by knitting together meagre amounts of income and livelihood derived from various tasks for cash in small towns and larger centres, mutual support networks and, increasingly, social assistance programmes. The directions of livelihood change described above, with diverging pathways apparent for those moving up, moving out, hanging in and dropping out, are changing the profiles of poverty and vulnerability in the rangelands, as described below.

## Impacts of change on poverty and vulnerability in the rangelands

Ongoing structural changes in rangeland Eastern Africa, and the paradoxical dynamics these have generated, have resulted in varied outcomes for inhabitants of the region, both within and across different sub-regional pastoral areas. In this setting, vulnerability is not something static but, rather, is a dynamic and constantly changing state relating to the very different livelihood mixes that are now apparent. High livestock losses coupled with demographic growth have changed the distribution of poverty, with per capita livestock holdings in a long decline (Lybbert et al. 2004; Desta et al. 2008; Teshome and Bayissa 2014). The impacts of this are apparent across the region in a downward shift in the income of different households, with growing numbers of people becoming destitute, or living in poor or middle-income households, and fewer classed as wealthy (Morton 2006 in Desta et al. 2008: 5). With livestock poverty deepening for many rangeland inhabitants, there has also been a shift towards keeping livestock in smaller herds that can be easily disposed of to meet cash needs. Both wealth and income inequalities are widening between those who are moving up and out and the others who are just about remaining within or have exited from a pastoralist way of life. Per capita income is highest for those in places with higher mobility and resource access alongside good market development (McPeak et al. 2012). Further, pastoralists who move appear to be significantly better off than ex-pastoralist sedentary farmers in the same region (especially farmers without irrigation) (Headey et al. 2012).

While many rangeland inhabitants are being pushed out of pastoralism, options for people to return to productive livestock-keeping are shrinking, as well. Evidence points to increased impoverishment, malnutrition and destitution of pastoralists who settle (Adano and Witsenburg 2005; Fratkin and Roth 2005). People in this group experience the highest levels of poverty and greatest challenges for achieving a secure livelihood. They depend on low-return activities that are vulnerable to shocks, such as the collection and sale of fodder, charcoal burning or harvesting and selling fuelwood. For most of those who have exited livestock production, diversification is about surviving, with limited capacity to scale up petty cottage industries, fuelwood or charcoal production (Headey et al. 2012) and only meagre incomes from the range of survival work that people engage in (Devereux 2006). Many who have diversified seek to keep a few animals when conditions permit. Having a small herd is disadvantageous, as small herds that are milked intensively experience lower calf growth and survival when pasture is scarce (Western and Nightingale 2004). This also makes it difficult for pastoralists to ‘build back’ herds. In contrast, wealthier households can leave more milk for livestock to consume, resulting in better calf health and more resilient herds (Holden et al. 1991 in McPeak and Little 2014: 56). These dynamics are often echoed in the relatively inferior nutrition status of settled households who do not keep herds or only a few livestock. Even if households build up a small herd, sustaining a mobile way of life in the rangelands is becoming increasingly difficult as land is fenced off, privatised and fragmented, as explored in the cases below. These changes in the rangelands are complicating pathways out of poverty, with opportunities diminishing for building herds and finding grazing.

While a large, and arguably growing, proportion of rangeland inhabitants struggle to make a living outside of or on the edge of pastoralism, some communities and individuals benefit greatly from expanding trade, marketing and opportunities for commercialisation. Wealth is becoming even more concentrated among the better-off who are well-positioned to move up into high-value livestock marketing and commercialisation. According to Aklilu and Catley (2009), the wealth generated from livestock marketing largely benefits those at the ‘sharp end of the business’ (exporters, ranchers, feedlot operators and butcheries) who are able to make the most out of both domestic and export livestock markets, and traders who can navigate the risky routes can also generate considerable relative wealth. These benefits do not reach those who are hanging in with small herds or those dropping out (the poorest households who need to build up their herds—if they still have animals at all), who are unable to take advantage of high-value marketing opportunities. In Borana, Aklilu and Catley found that ‘the middle and better off income groups also sold respectively six and twelve times more sheep and goats than the very poor’ (2009, p. 15).

The dynamics of intensifying commercialisation in the region, occurring alongside large new infrastructural and agricultural investments, are exclusionary. Although recent investments in roads represent a welcome renewal of interest by states in pastoral areas and an opportunity to improve the accessibility of markets and services for rangeland dwellers, the outcomes of capital projection in drylands can be ambiguous for smaller-scale livestock units and the small town poor. McPeak and Little (2014: 67) observe that, ‘While transport improvements can create new opportunities for more price responsive marketing and value added processing, it is by no means clear that the benefits to the majority of dryland residents will outweigh the costs, if current trends continue.’ Recent large-scale investments might lift the livelihoods of dryland populations; equally, they could constitute a new type of stressor in places where investment hastens land and resource grabbing, creating new restrictions on resource access (Lind et al. 2020).

Thus, evolving livelihoods in pastoral and post-pastoral rangelands are associated with significant changes in the profiles of poverty and vulnerability alike. New livelihood mixes entail very different options and opportunities for rangeland populations to manage uncertainties—both longstanding and new—and possibilities for reducing poverty and vulnerability. Impacts are non-linear and complex, with new patterns of social differentiation emerging as well as changes in gender and generational dynamics. As the ways of making a living shift in a multitude of directions, and diverse forms of accumulation emerge, gender and age norms are destabilised (Wangui 2014). Both opportunities and tensions arise as negotiations ensue around demands for labour as well as control of new wealth. The following section examines five rangeland settings where structural change has generated new differentiation and vulnerability while also challenging long-established ways of managing uncertainty.

## Change and differentiation across five rangeland contexts

### Somali Region (Eastern Ethiopia)

The Somali Region of Ethiopia is part of a wider ‘Somali export zone’ crossing into Somaliland and Puntland. Here, livelihoods have evolved from customary mobile pastoralism (though still involving marketing and trade) towards commercialised forms of livestock keeping that feed export markets (Eid 2014); others have exited into low-return economic activities. The estimated value of the regional trade in livestock and meat was US$1 billion for the Horn in 2010 (Catley et al. 2013). In 2014, the value of livestock going through Somali ports was close to 600 million USD in 2014 (FSNAU 2015) and about 50–65% of this is believed to originate from Somali Region of Ethiopia (Oxfam 2011). Even before infrastructural improvements in eastern Ethiopia’s lowlands and adjacent areas of northern Kenya, the region was connected to a larger regional livestock marketing and trade. In the late 1990s, the annual value of livestock going through the Somali ports of Berbera and Bossaso was estimated to be more than US$120 million, 80% of which came from the Somali Region (Shank 1997). Much of this was informal, and the volume annually of informal cross-border livestock trade accounts for considerably more trade than the official export trade (McPeak and Little 2006). The informal trade between Ethiopia and Somaliland feeds approximately 50% of the small stock exported from Berbera and Bossaso, most of which are sourced from eastern Ethiopia’s lowlands (Majid 2010).

Livestock commercialisation in the region has happened alongside processes of sedentarisation and the rapid growth of towns, both large and small (Korf et al. 2015). Although more recent data is not available, by 2007, the population of the region’s largest town, Jigjiga, had grown to more than 275,000 according to data from Ethiopia’s census (Gebreyesus 2019). It is thought to be one of the country’s fastest growing towns, with an annual average growth rate of more than 10%. Wealth differentiation and food aid incentives have led to rapid growth in the size of other towns in the region, with Kabri Dehar growing to a reported 60,000 people, for example (Goyder and Mathys 2012: 11). Urban growth is a manifestation of livelihood trends and economic change and diversification, with a growing proportion moving into livelihoods that do not depend on having herds. Also, as the population tilts to urban areas, this potentially represents a changing relationship between access to and use of natural resources and will affect the demand-supply relationship in agriculture as fewer people keep livestock and growing urban populations require feeding. Pastoralists have also responded to increasing domestic demands associated with the high and increasing human populations in urban areas, and rising purchasing power among some consumers (Abdullahi et al. 2013; Aklilu and Catley 2014; McPeak and Little 2006). Examples include the establishment and spread of camel micro-dairying operations by Gode town dwellers over the past 20 years, who supply both local markets and the Somali market in Addis (Abdullahi et al. 2013). Notably, women dominate milk sales in many areas; the commodification of milk has contributed to changing social norms and the elevated status of women as breadwinners (Pearson and Schmidt 2018; Sadler et al. 2012).

An economic rush in Somali Region, incentivised by transnational marketing networks, has seen the rapid commodification of pastoral resources such as charcoal, water points and cash crops (Korf et al. 2015). In Harshin, historically an important drought grazing reserve that lies on a strategic trekking route for livestock being exported through Berbera, there has been a near total privatisation of grazing areas and water as the rangeland was carved into household plots for farming and private grazing (Flintan et al. 2011). The uncontrolled expansion of permanent water points has encouraged sedentarisation and rangeland fragmentation, as well. More than 500 boreholes were constructed in a 20-year period up to 2010, as well as hundreds of water harvesting structures. Thus, livelihood changes in this region are indicative of the widening inequality gap between those who have enjoy greater access to high-value markets and the much larger proportion of the population that does not, and is exiting into low-value alternatives in and near to towns and urban centres. Still others are moving out into activities that do not depend on having herds but that still connect to the pastoral economy.

### Borana (southern Ethiopia)

Diverging livelihood pathways are also evident in the Borana zone of southern Ethiopia. Many pastoralists in this region have shifted from customary pastoralism into smaller-scale commercialised livestock-keeping, most recently of goats and sheep, while many have turned to rain-fed cultivation of small plots (McPeak et al. 2012; Tilahun et al. 2017). Like Somali Region, patterns of livelihood change in Borana have been influenced by processes of larger-scale livestock commercialisation and increasing exports of cattle and camels to markets in the Arabian Peninsula (Little et al. 2015; Mahmoud 2010). Many have been pushed out of livestock-keeping (Homann et al. 2008; Teshome and Bayissa 2014), finding work in providing casual labour, or selling wood, water and charcoal in and near small towns (Desta et al. 2008; Doyo et al. 2018). Over the past three decades, small towns have expanded across the region, and approximately 16% of the population now reside in 19 regional centres (CSA 2008). The pull factors towards towns and larger centres include work opportunities, access to public services, particularly education, and better living conditions. Though stockless, drop-outs remain closely tied to livestock-keeping in the region through their livelihood activities and social ties, and many still identify as ‘pastoralists’ (Teshome and Bayissa 2014). Men are getting involved in brokering, trekking and farming and women in fuel wood collection, passing contraband goods, local brewery and work as housemaids (ibid.). Little et al. (2001) found that after adopting a settled lifestyle, low-income women took on milk and vegetable trading, making handcrafts, brewing and waged labour, whereas wealthier women relied more on income from livestock, milk and the sale of ghee.

Structural change in Borana traces back several decades, at least to the 1970s when interventions by the state to increase rangeland productivity, most notably the construction of water ponds and a ban on customary practices of burning pastures (Angassa and Oba 2008), in conjunction with the promotion of crop cultivation and settlement, set in motion processes of degradation and impoverishment that continue to unfold. Large-scale bush encroachment resulting from the ban on pasture burning reduced available grazing. The expansion of crop cultivation has also reduced communal grazing areas (Tache 2013; Berhanu et al. 2007). Borana increasingly turned to crop cultivation following the 1984–1985 drought, when many impoverished households settled in areas around deep wells. Other changes in the rangelands have stemmed from the expropriation of land by the state for ranches and other large-scale investments, such as in the Liban plain (Tache 2013).

Borana responses to these pressures include the fencing of communal pastures reserves, or *kallo* (Homann et al. 2008; Tache 2013). While adopted as a response to reductions in grazing, the fencing of *kallo* has contributed to rangeland fragmentation. Comparisons are to be found with other rangeland settings in Eastern Africa, where sedentarisation and enclosure have resulted from a combination of state intervention and local efforts to enclose and commodify the commons (Korf et al. 2015). A land rush continues in Borana as those with connections to the regional political administration and urban businesspeople acquire land to establish ranches and for other individual uses (Coppock et al. 2018). This magnifies social differentiation as those with either influence and/or capital claim private land—either in ecologically superior pockets or positioned near new infrastructure and growing towns rangelands (Kamara et al. 2004; Homann et al. 2008; Aklilu and Catley 2009).

Diversification among better-off pastoralists is an increasingly important livelihood strategy whereby placing a family member in waged employment outside of pastoralism, and the rangeland provides capital for reinvesting in the livestock sector (McPeak and Little 2006). Over the past two decades, Borana pastoralists have become a major supplier of cattle destined for domestic and international markets (Coppock et al. 2012). Exports of livestock supplied by Borana began to expand from the early 2000s and particularly after 2009 when Saudi Arabia lifted the ban on livestock imports from the Horn of Africa. This coincided with the Government of Ethiopia’s official encouragement of the livestock sector through investments in quarantine facilities, feedlots and modern market yards as well as trade agreements with importing countries. Informal livestock trade, which had flowed from Borana into Kenya and Somalia during the Saudi livestock ban, reversed during this period as official exports of livestock and livestock products experienced double digit growth through 2010 (Aklilu and Catley 2009; Debsu 2013; Waktole 2014). Since then, exports of cattle have slowed and even dipped since 2014.

A complex range of people and processes, from the livestock owners to small-scale collectors/traders (*bittu*—many who are young men), brokers, larger traders, transporters, hotels and restaurants, are involved in the Borana cattle value chain. However, the benefits of cattle exports are unequally distributed, with profits concentrated among large-scale traders and exporters who are mostly from Ethiopia’s highlands. Borana herders themselves receive only a minimal percentage of the overall profit that the animal achieves, compared to other actors in the value chain. Costs of negotiating the long chain are prohibitive for many. Access to capital is key for hiring transport. However profitable, these long value chains are risky: the demand for livestock is unpredictable, prices fluctuate and there is substantial reliance on informal credit. Thus, like Somali Region in Ethiopia, livelihood changes in Borana in recent decades are characterised by a divergence between those able to seize opportunities in higher-value livestock marketing, and subsequently to invest in other new economic opportunities present around growing towns, and those who struggle to get by with smaller herds and diminished access to key grazing areas. In this setting, managing uncertainty is becoming ever more determined by wealth and access to economic opportunities in livestock-keeping and beyond. This has implications not only for class but also for gender and generation as the positions and roles of women and young people shift alongside diversifying livelihoods.

### South Rift valley (Kenya)

Examples abound in Eastern Africa of land and resource grabbing in the rangelands, as seen in Somali Region and Borana, or of pastoralists making ill-informed sales of individual land holdings. The commercialisation of land, rather than livestock, has changed the face of pastoralism in Kenya’s south Rift Valley, as many Maasai seek alternatives to livestock keeping. The establishment of group ranches by the state and the World Bank in the 1970s set in motion the individualisation of land tenure. Group ranch members were persuaded to agree to subdivision, despite the negative impact that it would have on customary methods of livestock-keeping, rendering these unviable (Mwangi 2007a). Instead, many opted for individual title deeds to a smaller area of land (Mwangi 2007b). Subdivision of Maasai areas led to increased settlement and loss of mobility for the herds (Kimani and Pickard 1998). The result was increased land degradation, and lower productivity for livestock rearing in these areas, increasing the Maasai’s vulnerability to drought (Thornton et al. 2006; Western and Nightingale 2004). While overall cattle and shoat numbers rose between 1973 and 2001, per capita holdings had fallen to just four in the 1980s, a trend that has continued (Western and Nightingale 2004). Some Maasai moved into permanent farming, not necessarily to become full-time farmers but as a way of rebuilding herds. In Loitokitok Division of Kajiado, the land under till expanded from 7500 ha to almost 30,000 between 1973 and 2000 (Campbell et al. 2003 in Wangui 2008: p. 372). Spurred by the area’s proximity to Nairobi and the presence of speculators looking to profit from the city’s expansion, others sold their plots to investors (Rutten 1992). Land subdivision and sedentarisation has also resulted in a decline in the size of household herds, something that is more pronounced among more sedentarised pastoralists (Kimiti et al. 2018). Immigration of livestock into these areas can also put pressure on grazing resources and increase herd losses, even in the absence of drought conditions (Nkedianye et al. 2011).

As the carving up of rangelands erodes the availability of resources for livestock-keeping, many are left worse off and the transition into a peri-urban frontier is accelerated. A small minority have, however, benefitted immensely from the sub-division of rangelands and conversion of key resources into other uses, including the establishment of flower farms supplying European markets.

Changes in pastoralist systems also have a huge impact on women and the way that roles are apportioned by gender within households (Wangui 2014). Sedentarisation and an increase in zero-grazing strategies to sustain and improve animal breeds have resulted in livestock being kept close to home. As more children and young people start to attend school, households experience labour shortages. Women, therefore, bear an increased burden for fodder and manure collection, milking and selling of milk and grazing small stock (Wangui 2008: p. 373).

Maasai communities have, over the past several decades, responded to the threats posed by further land alienation and the decline of pastoralism. Some responses are based in tradition, such as a shift to small stock rather than cattle to graze on more degraded areas and increase livestock drought resilience (Western and Nightingale 2004). Other responses are more market-oriented: milk and livestock sales and cultivation on partially subdivided ranches. The Maasai have also turned to wildlife-based industries, especially tourism. Wildlife migrates in and out of parks on to community lands, and wildlife numbers in the national parks have declined (Western et al. 2009). Much of Kenya’s wildlife is outside its national parks, on community lands or private ranches. Communities (especially Maasai communities) have set up their own conservancies to protect wildlife, and engage in ecotourism businesses, either on their own or in partnership with businesses in the private sector. However, the steep decline in wildlife populations in subdivided areas has serious implications for conservation and wildlife tourism and these newly established business opportunities (Groom and Western 2013).

Pastoralists are aware of the changes occurring in the rangelands, and the role of sedentarisation in reducing available grazing, and in causing land degradation (Kimiti et al. 2016). Privatisation has meant that decisions over land use and access to grazing are increasingly made by individuals. In the traditional context, such decisions were made communally. The new arrangements can threaten social bonds and networks, and access to resources. However, many Maasai communities in the south Rift are looking at ways to strengthen social capital and provide mutual assistance and access to resources on a wider scale. They have turned to each other, as well as to other institutions to join up as groups and share pastures, water, wildlife areas and other amenities. To this end, they have formed groups such as SORALO (South Rift Association of Land Owners) or ATGRCA (Amboseli/Tsavo Group Ranches Conservation Association). The latter has joined up group ranches and formed 17 conservancies in the Amboseli Ecosystem Trust. Together with the Kenya Wildlife Service and other conservation organisations, they formulated the Amboseli Ecosystem Management Plan, which addresses ecosystem threats, as well as opportunities such as livestock development, rangeland and water management, new enterprises and urbanisation. An overarching aim is to maintain habitat connectivity (for wildlife and livestock). SORALO incorporates 16 group ranches, over an area of 15,000 km^2^. SORALO, while maintaining land security for its members, aims to promote coexistence with wildlife and open rangelands alongside some agricultural activities and ecotourism (Western et al. 2020). As well as strengthening traditional kinds of resource management, south Rift communities are coming up with new strategies to counter fragmentation of their grazing areas and loss of pastoral livelihoods by grouping together and collaborating on sharing knowledge and resources.

### Karamoja (north-eastern Uganda)

Several communities in Karamoja in north-eastern Uganda are ‘hanging in’ transhumant pastoralism and small-scale agro-pastoralism. Livestock generate products—milk, blood and live and dead animals—as well as ploughing services with an estimated value of US$323 million in 2018–2019 (Behnke and Arasio 2019: 11), testament to the overwhelming economic importance and benefit of livestock in Karamoja up to now. Yet, many have also exited livestock keeping and are surviving through their involvement in a range of tasks-for-cash activities through which they generate a meagre income to support their livelihoods. A growing number of Karamojong families are now settled, stockless, making a living through different means, including small-scale farming and peasant work (Caravani 2019). What used to be ad hoc and short-term coping strategies, such as casual labour, brewing and firewood collection, became fundamental and long-term adaptive activities (Stites et al. 2007; Dancause et al. 2010). For instance, the function of beer as an item with exclusive important cultural and exchange-gift values has been commoditised (Mkutu 2008; Caravani 2017).

In the past, various non-livestock-related activities were carried out solely by women, as is farming and casual labour. Following a long period of sedentarisation and livestock dispossession, tasks-for-cash activities have become key survival work for women and men alike. Division of labour to produce these commodities is highly gendered; charcoal is exemplary, whereby the cutting of big trees is done by men while the burning and trading are generally done by women.

The expansion of petty trade and associated growth of small trading centres has provided new work opportunities for young men in butchering, unloading buses/lorries, brick making and construction and for women in domestic work (Caravani 2019; Stites et al. 2014). While brewing is a lucrative activity for women, their other work is generally less remunerative than men’s work (Stites and Huisman 2010). Livelihood diversification is not only about leaving pastoralism, but rather many use it as a way to remain in or move back into livestock keeping, as evidenced by migrants in towns who sustain links to family members and friends in rural areas (Stites et al. 2014). The latter group generally consist of a few large families whose members straddle both town and rural settings, reflecting a livelihood strategy that combines wealth generated from livestock-keeping with income streams off the rangelands. These large families are oftentimes among the better-off (Caravani 2019).

In Karamoja, several groups began settling in the more ecologically fecund south-western fringes of the region in the early 1980s, as famine and growing insecurity diminished herds. Like elsewhere in the region, some Karamojong were encouraged to settle by the introduction of agricultural schemes in more fertile areas as well as through the provision of food relief in schools and health centres (Cisternino 1985). This period signalled a growing reliance on non-state actors such as humanitarian organisations and religious groups that provided basic social assistance (Caravani 2017). These actors promoted Western social values and a different economic production system, thus influencing transitions away from pastoralism (Caravani 2019).

Since 2006, forced disarmament as a form of renewed state intervention in the region has resulted in the depletion of herds for many (FAO 2014). It is enjoined by a continuing promotion by the state of settled farming as a ‘civilised’ alternative to pastoralism. Pastoralism was condemned by government officials on several occasions. An uneasy peace has held since the disarmament campaign, which has marked a period of further deep social and political change.

In recent years, local capital and state investment, especially on roads and power infrastructures, have encouraged more dynamic growth in small towns. In Karamoja, rental and leisure markets have expanded as better-off Karamojong construct small hotels, housing and restaurants for recent migrants from within and outside the region. Up to now, many young Karamojong continue to seek opportunities outside of livestock-keeping, either in agriculture in more fertile areas such as Karenga, Abim, Namalu and Iriiri (Caravani 2019), or in towns (Caravani 2017; Stites et al. 2014), however with limited success. NGOs and civil service jobs are the best available options for a few politically connected families.

In sum, many former transhumant pastoralists are in fact not successfully transforming into wage labourers, petty traders and farmers, given the structural lack of opportunities and the low productivity of land for agriculture. The rise of non-livestock activities, such as charcoal production, brewing and causal labour, suggests that Karamojong are resorting to a mixture of both market-oriented and subsistence-oriented production systems to survive in the absence of reliable livelihood opportunities. The paradox is that this pastoral transition has been encouraged by humanitarian actors and religious organisations and endorsed by the state, the result of which is precarious livelihoods that are increasingly vulnerable to shocks.

### Northern Bahr el Ghazal (South Sudan)

With an estimated 1.6 million head of cattle, northern Bahr el Ghazal is home to South Sudan’s largest livestock population and an estimated 13% of the country’s overall total (Aklilu et al. 2016: 30). Bordering Sudan to the north, the region is well-endowed with small rivers and streams as well as fecund rangelands, which sustain a mix of livelihoods based on livestock-keeping and cultivation. However, decades of war and displacement have had profound consequences for livelihood practices, the household economy and access to markets (Aklilu et al. 2016). Conflict has resulted in declining per capita livestock holdings and along with this, worsening vulnerability, poverty and food insecurity (Aklilu et al. 2016; Deng 2008). Thus, while many ‘hang in’ herding and subsistence farming, the profile of these activities has changed significantly. Further, the balance between farming and herding has shifted, with at least some investing more in keeping livestock (Deng 2010). Still, customary forms of livestock-keeping in the region have been greatly affected by civil war, disease and the lack of an infrastructure and systems for providing rapid support.

Chronic insecurity in northern Bahr el Ghazal has meant that security considerations now determine livestock movements as much as the availability of water and grazing. Customary migration routes have been curtailed by war, pushing herders into precarious situations of seeking access to grazing in areas that are unfamiliar or less suited to the grazing needs of their herds. Insecurity has forced pastoralists to extend herd movements to more secure areas, including in the neighbouring states of Lakes, Warrap, Upper Nile and Greater Equatoria (FAO 2015). These movements, and the resulting intermingling of livestock from different areas, has contributed to the spread of livestock disease as well as renewed tensions. Both the spread of disease and restrictions on grazing access have contributed to losses of livestock, which in turn have consequences for health, nutrition and wellbeing. Furthermore, the earnings of women from milk sales have declined, with consequences for the food security and wellbeing of other family members. Still, despite tensions along the border between South Sudan and Sudan, pastoralists in Bahr el Ghazal have worked to develop peaceful relations with Arab herders and traders across the border to improve connections with wider livestock trades as well as access to basic commodities (Aklilu et al. 2016).

The impacts of ongoing conflict mean that many have fallen out of pastoralism and sought alternatives in other subsistence activities and survival work. For most, these options are located near towns, many of which have their origins as relief distribution centres and that continue to grow under the weight of people seeking assistance. Aweil, the state capital, has grown to more than 100,000 and now provides critical infrastructure and services for a far larger population beyond northern Bahr el Ghazal.

The cutting of trees is a key environmental concern as more shift to fuelwood harvesting and charcoal burning as alternatives to keeping livestock, as well as to meet increasing demand for fuel in growing towns and settlements. According to the 2009 National Budget Household Survey, 97% of the population uses firewood or charcoal as cooking fuel (USAID 2012). This situation is compounded by a lack of strong governance—both customary and state-led—that has led to unregulated and uncontrolled clearing of land to expand cultivation, for unlawful logging for timber exports, and as supplies for the construction industry (brick making and lumber).

Thus, recurrent insecurity and conflict constrain access to resources and markets that are needed for the wider emergence of ‘moving out’ or ‘moving up’ types of pastoralism. Instead, the region’s inhabitants persist with forms of livestock-keeping and farming that are lower risk but also low return. The drivers of changing rangeland livelihoods in northern Bahr el Ghazal are political rather than environmental. Prolonged conflict has militarised livelihoods, with young men increasingly mobilised to defend the political interests of ruling (and warring) elites rather than protect life and property in their communities, including livestock (Wild et al. 2018). The proliferation of small arms has distorted customary institutions, with the power of traditional authorities increasingly weakened by the emergence of new armed powers (Kuol 2017). Thus, chronic conflict is not only a manifestation of governance failures but also how states themselves undermine rural livelihoods by failing to create a conducive security environment (Kuol 2019). Without addressing these governance failures, conflict will continue to define how livelihoods in the region evolve.

### Summary

The very different livelihood trajectories in Eastern Africa’s rangelands, exemplified in the settings examined above, relate to varying access to markets and resources and the nesting of these in diverse political economies and ecological and socio-cultural systems. The cases described here show how situations of relative advantage and disadvantage have emerged around, and are themselves manifestation of, the paradoxical state of pastoral and post-pastoral rangelands. Some positions of relative advantage (or disadvantage) concern socially and culturally embedded social differences, such as gender, age and section/clan affiliation. Cultural and social norms in rangeland societies have shifted over a long period of livelihood transformation as women and young people assume new roles and positions. These are resulting in new claims to power within families as well as in politics and business at multiple levels of governance. Alongside longstanding social differences are other newly important lines of contrast defined by status, connections to political-administrative, business and other aid actors, and access to capital or weapons. While some have accumulated greater wealth in terms of livestock as well as income from an assortment of new complementary economic activities and investments, many continue to struggle with small herds (or have become stockless) and by necessity must balance a diverse portfolio of activities on and off the rangelands for their food security, nutrition and health. Thus, even while economic growth and complexity have accelerated, and rangeland elites enjoy newfound prosperity, the population requiring support to meet basic needs remains high and arguably is increasing in some places. Bouncing back to a pre-existing state is remote for most.

## Conclusions

The rangelands of Eastern Africa, and the diverse inhabitants of these areas, have experienced sweeping changes in access to both markets and natural resources, precipitating a number of paradoxical dynamics that have significant implications for addressing poverty and vulnerability as well as strengthening responses to uncertainty. Diverging livelihood pathways across and within various rangeland settings highlight the challenges of formulating approaches to reduce poverty and vulnerability. While there is a reasonable understanding of broad livelihood changes in the region, and emergent forms of pastoralism in evolving rangelands, individuals and groups who follow a similar pathway may nonetheless experience distinct advantages and disadvantages and, hence, require varying sorts of support. Many poverty reduction interventions in the rangelands advocate for diversification as a way of reducing risk and vulnerability. Yet, the livelihoods of many inhabitants are already highly diversified. Further, diversification-as-poverty-reduction runs counter to how many rangeland inhabitants have sought over time to secure better livelihoods, which is by investing in livestock-keeping when given the opportunity.

The situation in evolving pastoral and post-pastoral rangelands indicates the need to work with and through existing transformations. New livelihood mixes are already well- established, and they need to be better understood and supported. Evolving livelihoods in the region point to a future in which livestock-keeping will continue to have an important yet different role, with a greater emphasis on connecting livestock-keeping with running businesses, providing services in growing towns and supporting investments and specialisation in other non-livestock activities. Just as now, elements of pastoralism will entwine with a broad range of non-livestock livelihoods and productive activities existing in rangelands as well, which nonetheless may be associated with pastoralism through a variety of social and economic relationships. Social difference and differentiation are likely to expand even further as people not only seek different approaches to making a living but also people’s needs and uses of keeping livestock multiply.

Strengthening livelihoods in the rangelands requires a long-term perspective and interventions that are both multi-sectoral as well as multi-scalar. An obvious challenge is a significant gap in evidence and data across different systems and a distinct lack of longitudinal data to track change over time. While this article is limited to mapping changes over a 20-year period, an even deeper introspection can uncover more rooted dynamics that are critical to recognising longitudinal continuities as well as breaks with past patterns. A longer view is needed to overcome the tendency of more circumspective perspectives that might overplay or wrongly interpret shorter-term trends. Further, most existing survey data is incapable of describing or tracing the complexity of rangeland livelihoods (Randall 2015). An inherent bias of many research efforts is that they emphasise administrative units, thereby missing critical flows and connections across borders and groups, which may nonetheless be revealing of the ‘wiring’ of livelihoods and productive activity.

In other words, there is a substantial disconnect between literature describing changes and the ability of data to represent evolving livelihoods. A better understanding of livelihood configurations in these places requires more extensive data collection over time as well as more precise insights into what is happening for people moving along different pathways (moving up, moving out, hanging in and dropping out). The next step in this work will be to bring different types of methods, data and analysis together to provide a grounded understanding of trajectories of change for different categories of pastoralists. This work can uncover the implications and requirements for addressing poverty and vulnerability in a context of continuing dynamic change.

## Data Availability

Data sets that were identified as part of background research, a coded catalogue of secondary sources that were reviewed and historical timelines that were prepared for different pastoral systems in Eastern Africa can be found here: https://opendocs.ids.ac.uk/opendocs/handle/123456789/12082.

## Abbreviations

ATGRCA: Amboseli/Tsavo Group Ranches Conservation Association
FAO: Food and Agriculture Organization
FSNAU: Food Security and Nutrition Analysis Unit
NGO: Non-governmental organisation
SORALO: South Rift Association of Land Owners
USAID: United States Agency for International Development

## Footnotes

1 For a detailed explanation of research method, see Lind et al. (2016).
